# Bis(nitrato-κ*O*)[(*S*)-2-(pyrrolidin-2-yl)-1*H*-benzimidazole]cadmium(II)

**DOI:** 10.1107/S1600536808006454

**Published:** 2008-07-09

**Authors:** Wei Dai, Da-Wei Fu

**Affiliations:** a Ordered Matter Science Research Center, College of Chemistry and Chemical Engineering, Southeast University, Nanjing 210096, People’s Republic of China

## Abstract

The title compound, [Cd(NO_3_)_2_(C_11_H_13_N_3_)_2_], was synthesized by hydro­thermal reaction of Cd(NO_3_)_2_ and *S*-2-(pyrrolidin-2-yl)-1*H*-1,3-benzimidazole. The Cd atom lies on an inversion centre. The distorted octa­hedral Cd environment contains two planar *trans*-related *N*,*N*-chelating *S*-2-(pyrrolidin-2-yl)-1*H*-1,3-benzimidazole ligands in one plane and two monodentate nitrate ligands. N—H⋯O hydrogen bonds involving a nitrate O atom build up an infinite chain parallel to the *a* axis.

## Related literature

For physical properties such as fluorescence and dielectric behaviors of metal–organic coordination compounds, see: Aminabhavi *et al.* (1986 ▶); Ye *et al.* (2008 ▶).
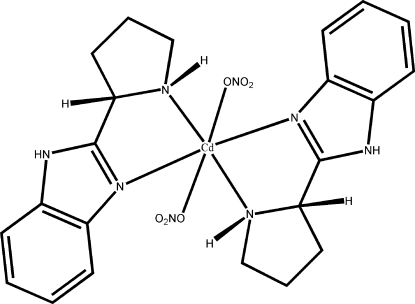


## Experimental

### 

#### Crystal data

[Cd(NO_3_)_2_(C_11_H_12_N_3_)_2_]
*M* *_r_* = 610.91
Triclinic, 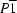

*a* = 8.1487 (16) Å
*b* = 9.1459 (18) Å
*c* = 9.7439 (19) Å
α = 111.67 (3)°
β = 112.32 (3)°
γ = 93.80 (3)°
*V* = 606.0 (2) Å^3^
*Z* = 1
Mo *K*α radiation
μ = 0.96 mm^−1^
*T* = 293 (2) K
0.12 × 0.10 × 0.06 mm

#### Data collection

Rigaku Mercury2 diffractometer
Absorption correction: multi-scan (*CrystalClear*; Rigaku, 2005 ▶) *T*_min_ = 0.889, *T*_max_ = 0.944
6172 measured reflections
2692 independent reflections
2258 reflections with *I* > 2σ(*I*)
*R*_int_ = 0.057

#### Refinement

*R*[*F* ^2^ > 2σ(*F* ^2^)] = 0.053
*wR*(*F* ^2^) = 0.114
*S* = 1.07
2692 reflections
169 parameters
H-atom parameters constrained
Δρ_max_ = 0.69 e Å^−3^
Δρ_min_ = −0.45 e Å^−3^

### 

Data collection: *CrystalClear* (Rigaku, 2005 ▶); cell refinement: *CrystalClear*; data reduction: *CrystalClear* program(s) used to solve structure: *SHELXS97* (Sheldrick, 2008 ▶); program(s) used to refine structure: *SHELXL97* (Sheldrick, 2008 ▶); molecular graphics: *SHELXTL* (Sheldrick, 2008 ▶); software used to prepare material for publication: *SHELXTL*.

## Supplementary Material

Crystal structure: contains datablocks I, global. DOI: 10.1107/S1600536808006454/dn2315sup1.cif

Structure factors: contains datablocks I. DOI: 10.1107/S1600536808006454/dn2315Isup2.hkl

Additional supplementary materials:  crystallographic information; 3D view; checkCIF report

## Figures and Tables

**Table 1:** Hydrogen-bond geometry (Å, °)

*D*—H⋯*A*	*D*—H	H⋯*A*	*D*⋯*A*	*D*—H⋯*A*
N3—H3*B*⋯O1^i^	0.91	2.21	2.975 (7)	141
N1—H1*A*⋯O2^ii^	0.86	2.03	2.889 (5)	174

Symmetry codes: (i) 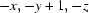; (ii) 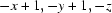.

**Fig. 1.**
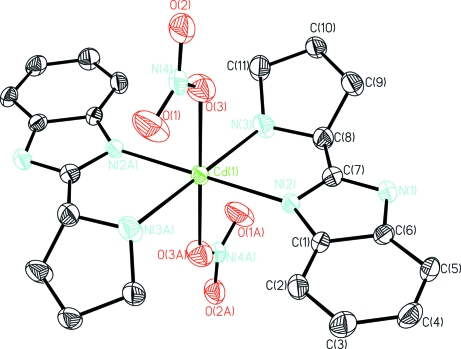
A view of the title compound with the atomic numbering scheme. Displacement ellipsoids were drawn at the 30% probability level.

**Fig. 2.**
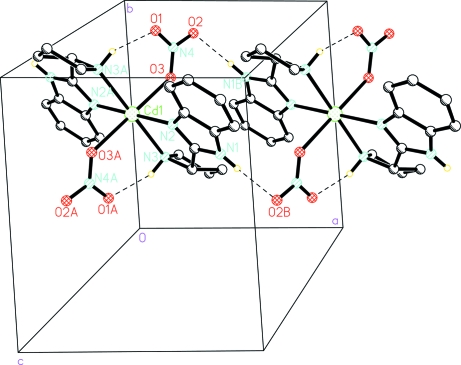
The crystal packing of the title compound viewed along the c axis and all hydrogen atoms not involved in hydrogen bonding (dashed lines) were omitted for clarity.

## supplementary crystallographic information

### Comment

Metal-organic coordination compounds provide a class of complexes displaying interesting chemical and physical properties such as fluorescence and dielectric behaviors (Aminabhavi *et al., *1986; Ye *et al.*, 2008). There has been very strong interest in employing crystal-engineering strategies to generate desirable materials by the hydrothermal reaction. Here we report the synthesis and crystal structure of the title compound Nitrate-(*S*-2-(pyrrolidin-2-yl)-1*H*-benzo[*d*]imidazole)-Cadmium).

In the title compound, the cadmium atom lies on an inversion centre. The distorted octahedral Cd environment contains two planar *trans*-related N,*N*-chelating S-2-(pyrrolidin-2-yl)-1*H*-benzo imidazole in one plane and two monodentate nitrate (Fig. 1). N—H···O hydrogen bonds involving one O atom of the nitrate build up an infinite chain developing parallel to the *a* axis (Table 1).

### Experimental

The homochiral ligand *S*-2-(pyrrolidin-2-yl)-1*H*-benzo[*d*]imidazole was synthesized by reaction of *S*-pyrrolidine-2-carboxylic acid and benzene-1,2-diamine according to the procedure described in the literature(Aminabhavi, *et al.*(1986)). A mixture of *S*-2-(pyrrolidin-2-yl)-1*H*-benzo[*d*]imidazole(0.1 mmol) and Cd(NO_3_)_2_ (0.1 mmol) and water (1 ml) sealed in a glass tube were maintained at 70 °C. Crystals suitable for X-ray analysis were obtained after 3 days.

### Refinement

Positional parameters of all the H atoms bonded to C or N atoms were calculated geometrically and were allowed to ride on the C atoms to which they are bonded, with *U*_iso_(H) = 1.2Ueq(C or N).

### Crystal data

**Table:**

[Cd(NO_3_)_2_(C_11_H_12_N_3_)_2_]	*Z* = 1
*M**_r_* = 610.91	*F*_000_ = 310
Triclinic, *P*1	*D*_x_ = 1.674 Mg m^−^^3^
Hall symbol: -P1	Mo *K*α radiation λ = 0.71073 Å
*a* = 8.1487 (16) Å	Cell parameters from 2061 reflections
*b* = 9.1459 (18) Å	θ = 3.3–27.5º
*c* = 9.7439 (19) Å	µ = 0.96 mm^−^^1^
α = 111.67 (3)º	*T* = 293 (2) K
β = 112.32 (3)º	Prism, colorless
γ = 93.80 (3)º	0.12 × 0.10 × 0.06 mm
*V* = 606.0 (2) Å^3^	

### Data collection

**Table:**

Rigaku Mercury2 diffractometer	2692 independent reflections
Radiation source: fine-focus sealed tube	2258 reflections with *I* > 2σ(*I*)
Monochromator: graphite	*R*_int_ = 0.057
Detector resolution: 13.6612 pixels mm^-1^	θ_max_ = 27.3º
*T* = 293(2) K	θ_min_ = 3.3º
CCD profile fitting scans	*h* = −10→10
Absorption correction: multi-scan(CrystalClear; Rigaku, 2005)	*k* = −11→11
*T*_min_ = 0.889, *T*_max_ = 0.944	*l* = −12→12
6172 measured reflections	

### Refinement

**Table:**

Refinement on *F*^2^	Secondary atom site location: difference Fourier map
Least-squares matrix: full	Hydrogen site location: inferred from neighbouring sites
*R*[*F*^2^ > 2σ(*F*^2^)] = 0.053	H-atom parameters constrained
*wR*(*F*^2^) = 0.114	*w* = 1/[σ^2^(*F*_o_^2^) + (0.0464*P*)^2^ + 0.245*P*] where *P* = (*F*_o_^2^ + 2*F*_c_^2^)/3
*S* = 1.07	(Δ/σ)_max_ < 0.001
2692 reflections	Δρ_max_ = 0.69 e Å^−^^3^
169 parameters	Δρ_min_ = −0.44 e Å^−^^3^
Primary atom site location: structure-invariant direct methods	Extinction correction: none

### Special details

**Table:**

Geometry. All e.s.d.'s (except the e.s.d. in the dihedral angle between two l.s. planes) are estimated using the full covariance matrix. The cell e.s.d.'s are taken into account individually in the estimation of e.s.d.'s in distances, angles and torsion angles; correlations between e.s.d.'s in cell parameters are only used when they are defined by crystal symmetry. An approximate (isotropic) treatment of cell e.s.d.'s is used for estimating e.s.d.'s involving l.s. planes.
Refinement. Refinement of *F*^2^ against ALL reflections. The weighted *R*-factor *wR* and goodness of fit *S* are based on *F*^2^, conventional *R*-factors *R* are based on *F*, with *F* set to zero for negative *F*^2^. The threshold expression of *F*^2^ > σ(*F*^2^) is used only for calculating *R*-factors(gt) *etc*. and is not relevant to the choice of reflections for refinement. *R*-factors based on *F*^2^ are statistically about twice as large as those based on *F*, and *R*- factors based on ALL data will be even larger.

### Fractional atomic coordinates and isotropic or equivalent isotropic displacement parameters (Å^2^)

**Table:**

	*x*	*y*	*z*	*U*_iso_*/*U*_eq_	
Cd1	0.0000	0.5000	0.0000	0.03774 (18)	
O1	0.1413 (6)	0.8668 (6)	−0.0027 (6)	0.0919 (14)	
O2	0.2395 (5)	0.7989 (5)	−0.1864 (5)	0.0701 (11)	
O3	0.1342 (6)	0.6144 (5)	−0.1353 (5)	0.0730 (11)	
N4	0.1714 (5)	0.7596 (6)	−0.1065 (5)	0.0503 (10)	
N3	0.0853 (6)	0.2624 (5)	−0.1240 (5)	0.0585 (11)	
H3B	0.0050	0.1820	−0.1322	0.070*	
N2	0.2902 (4)	0.5267 (4)	0.1908 (4)	0.0376 (8)	
N1	0.5347 (5)	0.4236 (5)	0.2503 (5)	0.0470 (9)	
H1A	0.6040	0.3577	0.2384	0.056*	
C3	0.5789 (7)	0.8578 (6)	0.6093 (6)	0.0557 (13)	
H3A	0.5818	0.9564	0.6869	0.067*	
C5	0.7348 (6)	0.6476 (6)	0.5341 (5)	0.0453 (11)	
H5A	0.8365	0.6041	0.5576	0.054*	
C6	0.5793 (6)	0.5690 (5)	0.3855 (5)	0.0376 (9)	
C4	0.7314 (7)	0.7920 (6)	0.6443 (6)	0.0522 (12)	
H4A	0.8331	0.8476	0.7448	0.063*	
C7	0.3627 (6)	0.4036 (6)	0.1402 (5)	0.0444 (11)	
C2	0.4236 (6)	0.7797 (6)	0.4619 (6)	0.0487 (11)	
H2A	0.3221	0.8238	0.4398	0.058*	
C8	0.2668 (7)	0.2502 (6)	−0.0174 (6)	0.0524 (12)	
H8A	0.2472	0.1603	0.0108	0.063*	
C10	0.2213 (6)	0.1190 (6)	−0.2943 (6)	0.0543 (13)	
H10A	0.2635	0.1286	−0.3719	0.065*	
H10B	0.1804	0.0053	−0.3229	0.065*	
C9	0.3685 (7)	0.2075 (8)	−0.1197 (6)	0.0741 (18)	
H9A	0.4472	0.1382	−0.0909	0.089*	
H9B	0.4424	0.3042	−0.1055	0.089*	
C11	0.0726 (9)	0.2075 (9)	−0.2880 (6)	0.085 (2)	
H11A	0.0886	0.2992	−0.3131	0.102*	
H11B	−0.0463	0.1356	−0.3678	0.102*	
C1	0.4241 (6)	0.6332 (5)	0.3478 (5)	0.0360 (9)	

### Atomic displacement parameters (Å^2^)

**Table:**

	*U*^11^	*U*^22^	*U*^33^	*U*^12^	*U*^13^	*U*^23^
Cd1	0.0274 (3)	0.0437 (3)	0.0305 (3)	0.00864 (18)	0.00878 (18)	0.00795 (19)
O1	0.084 (3)	0.095 (3)	0.080 (3)	0.020 (3)	0.050 (3)	0.005 (3)
O2	0.073 (3)	0.080 (3)	0.092 (3)	0.040 (2)	0.048 (2)	0.057 (2)
O3	0.074 (3)	0.077 (3)	0.092 (3)	0.021 (2)	0.050 (2)	0.045 (2)
N4	0.032 (2)	0.071 (3)	0.046 (2)	0.022 (2)	0.0137 (18)	0.024 (2)
N3	0.043 (2)	0.060 (3)	0.048 (2)	0.012 (2)	0.016 (2)	0.003 (2)
N2	0.0310 (19)	0.044 (2)	0.0297 (17)	0.0114 (15)	0.0110 (15)	0.0098 (15)
N1	0.039 (2)	0.060 (3)	0.044 (2)	0.0233 (18)	0.0198 (18)	0.0208 (19)
C3	0.054 (3)	0.051 (3)	0.040 (3)	0.009 (2)	0.011 (2)	0.008 (2)
C5	0.034 (2)	0.060 (3)	0.041 (2)	0.015 (2)	0.010 (2)	0.027 (2)
C6	0.036 (2)	0.044 (2)	0.032 (2)	0.0081 (18)	0.0126 (19)	0.0183 (19)
C4	0.044 (3)	0.060 (3)	0.034 (2)	0.005 (2)	0.004 (2)	0.016 (2)
C7	0.043 (3)	0.054 (3)	0.035 (2)	0.017 (2)	0.017 (2)	0.018 (2)
C2	0.039 (3)	0.054 (3)	0.044 (3)	0.019 (2)	0.014 (2)	0.015 (2)
C8	0.050 (3)	0.055 (3)	0.044 (3)	0.020 (2)	0.017 (2)	0.015 (2)
C10	0.046 (3)	0.065 (3)	0.036 (3)	0.017 (2)	0.016 (2)	0.007 (2)
C9	0.044 (3)	0.108 (5)	0.038 (3)	0.023 (3)	0.013 (2)	0.002 (3)
C11	0.081 (4)	0.113 (5)	0.034 (3)	0.062 (4)	0.016 (3)	0.008 (3)
C1	0.031 (2)	0.044 (2)	0.033 (2)	0.0113 (18)	0.0107 (18)	0.0181 (19)

### Geometric parameters (Å, °)

**Table:**

Cd1—N2^i^	2.314 (3)	C3—H3A	0.9300
Cd1—N2	2.314 (3)	C5—C4	1.370 (7)
Cd1—N3^i^	2.359 (4)	C5—C6	1.391 (6)
Cd1—N3	2.359 (4)	C5—H5A	0.9300
Cd1—O3	2.448 (4)	C6—C1	1.409 (6)
Cd1—O3^i^	2.448 (4)	C4—H4A	0.9300
O1—N4	1.238 (5)	C7—C8	1.513 (7)
O2—N4	1.245 (5)	C2—C1	1.391 (6)
O3—N4	1.241 (5)	C2—H2A	0.9300
N3—C11	1.447 (7)	C8—C9	1.488 (7)
N3—C8	1.490 (6)	C8—H8A	0.9800
N3—H3B	0.9100	C10—C11	1.509 (7)
N2—C7	1.327 (6)	C10—C9	1.514 (7)
N2—C1	1.403 (5)	C10—H10A	0.9700
N1—C7	1.352 (6)	C10—H10B	0.9700
N1—C6	1.384 (6)	C9—H9A	0.9700
N1—H1A	0.8600	C9—H9B	0.9700
C3—C2	1.385 (7)	C11—H11A	0.9700
C3—C4	1.397 (7)	C11—H11B	0.9700
			
N2^i^—Cd1—N2	180.00 (18)	N1—C6—C1	105.2 (4)
N2^i^—Cd1—N3^i^	75.24 (13)	C5—C6—C1	122.3 (4)
N2—Cd1—N3^i^	104.76 (13)	C5—C4—C3	121.6 (4)
N2^i^—Cd1—N3	104.76 (13)	C5—C4—H4A	119.2
N2—Cd1—N3	75.24 (13)	C3—C4—H4A	119.2
N3^i^—Cd1—N3	180.0	N2—C7—N1	112.7 (4)
N2^i^—Cd1—O3	90.22 (13)	N2—C7—C8	125.9 (4)
N2—Cd1—O3	89.78 (13)	N1—C7—C8	121.4 (4)
N3^i^—Cd1—O3	94.44 (15)	C3—C2—C1	117.9 (4)
N3—Cd1—O3	85.56 (15)	C3—C2—H2A	121.1
N2^i^—Cd1—O3^i^	89.78 (13)	C1—C2—H2A	121.1
N2—Cd1—O3^i^	90.22 (13)	N3—C8—C9	106.3 (4)
N3^i^—Cd1—O3^i^	85.56 (15)	N3—C8—C7	111.2 (4)
N3—Cd1—O3^i^	94.44 (15)	C9—C8—C7	114.6 (5)
O3—Cd1—O3^i^	180.0	N3—C8—H8A	108.2
N4—O3—Cd1	126.5 (3)	C9—C8—H8A	108.2
O1—N4—O3	122.5 (5)	C7—C8—H8A	108.2
O1—N4—O2	118.7 (5)	C11—C10—C9	101.8 (4)
O3—N4—O2	118.9 (4)	C11—C10—H10A	111.4
C11—N3—C8	107.4 (4)	C9—C10—H10A	111.4
C11—N3—Cd1	122.4 (4)	C11—C10—H10B	111.4
C8—N3—Cd1	113.9 (3)	C9—C10—H10B	111.4
C11—N3—H3B	103.7	H10A—C10—H10B	109.3
C8—N3—H3B	103.7	C8—C9—C10	104.6 (4)
Cd1—N3—H3B	103.7	C8—C9—H9A	110.8
C7—N2—C1	105.2 (3)	C10—C9—H9A	110.8
C7—N2—Cd1	113.3 (3)	C8—C9—H9B	110.8
C1—N2—Cd1	141.5 (3)	C10—C9—H9B	110.8
C7—N1—C6	107.9 (4)	H9A—C9—H9B	108.9
C7—N1—H1A	126.1	N3—C11—C10	107.5 (4)
C6—N1—H1A	126.1	N3—C11—H11A	110.2
C2—C3—C4	121.5 (5)	C10—C11—H11A	110.2
C2—C3—H3A	119.2	N3—C11—H11B	110.2
C4—C3—H3A	119.2	C10—C11—H11B	110.2
C4—C5—C6	117.0 (4)	H11A—C11—H11B	108.5
C4—C5—H5A	121.5	C2—C1—N2	131.3 (4)
C6—C5—H5A	121.5	C2—C1—C6	119.7 (4)
N1—C6—C5	132.5 (4)	N2—C1—C6	109.0 (4)
			
N2^i^—Cd1—O3—N4	88.5 (4)	C2—C3—C4—C5	0.3 (8)
N2—Cd1—O3—N4	−91.5 (4)	C1—N2—C7—N1	−1.5 (5)
N3^i^—Cd1—O3—N4	13.3 (4)	Cd1—N2—C7—N1	177.7 (3)
N3—Cd1—O3—N4	−166.7 (4)	C1—N2—C7—C8	175.2 (5)
O3^i^—Cd1—O3—N4	−136 (100)	Cd1—N2—C7—C8	−5.6 (6)
Cd1—O3—N4—O1	−0.9 (6)	C6—N1—C7—N2	1.2 (5)
Cd1—O3—N4—O2	−179.9 (3)	C6—N1—C7—C8	−175.7 (4)
N2^i^—Cd1—N3—C11	51.1 (5)	C4—C3—C2—C1	−0.6 (8)
N2—Cd1—N3—C11	−128.9 (5)	C11—N3—C8—C9	7.3 (6)
N3^i^—Cd1—N3—C11	137 (16)	Cd1—N3—C8—C9	−131.4 (4)
O3—Cd1—N3—C11	−38.0 (5)	C11—N3—C8—C7	132.7 (5)
O3^i^—Cd1—N3—C11	142.0 (5)	Cd1—N3—C8—C7	−6.0 (5)
N2^i^—Cd1—N3—C8	−177.1 (3)	N2—C7—C8—N3	8.1 (7)
N2—Cd1—N3—C8	2.9 (3)	N1—C7—C8—N3	−175.4 (4)
N3^i^—Cd1—N3—C8	−91 (16)	N2—C7—C8—C9	128.7 (5)
O3—Cd1—N3—C8	93.8 (4)	N1—C7—C8—C9	−54.9 (7)
O3^i^—Cd1—N3—C8	−86.2 (4)	N3—C8—C9—C10	−26.8 (6)
N2^i^—Cd1—N2—C7	61 (100)	C7—C8—C9—C10	−150.1 (5)
N3^i^—Cd1—N2—C7	−178.9 (3)	C11—C10—C9—C8	35.0 (7)
N3—Cd1—N2—C7	1.1 (3)	C8—N3—C11—C10	15.2 (7)
O3—Cd1—N2—C7	−84.3 (3)	Cd1—N3—C11—C10	149.7 (4)
O3^i^—Cd1—N2—C7	95.7 (3)	C9—C10—C11—N3	−31.1 (7)
N2^i^—Cd1—N2—C1	−120 (100)	C3—C2—C1—N2	179.7 (5)
N3^i^—Cd1—N2—C1	−0.1 (5)	C3—C2—C1—C6	0.5 (7)
N3—Cd1—N2—C1	179.9 (5)	C7—N2—C1—C2	−178.0 (5)
O3—Cd1—N2—C1	94.5 (5)	Cd1—N2—C1—C2	3.2 (8)
O3^i^—Cd1—N2—C1	−85.5 (5)	C7—N2—C1—C6	1.3 (5)
C7—N1—C6—C5	178.6 (5)	Cd1—N2—C1—C6	−177.6 (3)
C7—N1—C6—C1	−0.3 (5)	N1—C6—C1—C2	178.8 (4)
C4—C5—C6—N1	−178.7 (5)	C5—C6—C1—C2	−0.3 (7)
C4—C5—C6—C1	0.0 (7)	N1—C6—C1—N2	−0.6 (5)
C6—C5—C4—C3	−0.1 (7)	C5—C6—C1—N2	−179.6 (4)

Symmetry codes: (i) −*x*, −*y*+1, −*z*.

### Hydrogen-bond geometry (Å, °)

**Table:**

*D*—H···*A*	*D*—H	H···*A*	*D*···*A*	*D*—H···*A*
N3—H3B···O1^i^	0.91	2.21	2.975 (7)	141
N1—H1A···O2^ii^	0.86	2.03	2.889 (5)	174

Symmetry codes: (i) −*x*, −*y*+1, −*z*; (ii) −*x*+1, −*y*+1, −*z*.
